# Impact of intraprocedural antiplatelet therapy on stent patency and safety after emergent intracranial stenting in acute ischaemic stroke: insights from the RESISTANT registry

**DOI:** 10.1093/esj/aakaf005

**Published:** 2026-01-01

**Authors:** Francesco Diana, Ameer E Hassan, Santiago Ortega-Gutierrez, Samantha Miller, Aaron Rodriguez-Calienes, Marta Olive Gadea, Johannes Kaesmacher, Adnan Mujanovic, Serdar Geyik, Songul Senadim, Mariangela Piano, Amedeo Cervo, Andrea Salcuni, Manuel Moreu, Alfonso López-Frías, Elena Zapata Arriaza, Asier de Albóniga-Chindurza, Mauro Bergui, Stefano Molinaro, João André Sousa, João Sargento-Freitas, Fábio Gomes, Andrea Alexandre, Alessandro Pedicelli, Paolo Machi, Jeremy Hofmeister, Luca Scarcia, Erwah Kalsoum, Jose Amorim, Torcato Meira, Leonardo Renieri, Francesco Capasso, Daniele G Romano, Eduardo Barcena, David Seoane, Mohamad Abdalkader, Piers Klein, Thanh N Nguyen, Catarina Perry, Isabel Fragata, Dileep R Yavagal, Jude H Charles, José Rodríguez, Pedro Vega, Atilla Ö Özdemir, Zehra Uysal, Stanislas Smajda, Sadiq Al Salman, Jane Khalife, Tudor Jovin, Francesco Biraschi, Francesca Ricchetti, Pedro Castro, Luis Albuquerque, Adnan H Siddiqui, Vinay Jaikumar, Pedro Navia, Nikolaos Ntoulias, Marios Psychogios, Mariano Velo, Joaquín Zamarro, Gonzalo de Paco, Yazan Ashouri, Mohammad AlMajali, Juan F Arenillas, Alicia Sierra, Michele Romoli, João Pedro Marto, Shadi Yaghi, Simone Peschillo, Marc Ribo, Alejandro Tomasello, Manuel Requena

**Affiliations:** Interventional Neuroradiology, Vall d'Hebron University Hospital, Barcelona, Spain; Vall d’Hebron Research Institute, Barcelona, Spain; Department of Life Sciences, Health and Health Professions, Link Campus University, Rome, Italy; Department of Neurology, Valley Baptist Medical Center, Harlingen, TX, United States; Department of Neurology, University of Iowa, Iowa City, IA, United States; Department of Neurology, Valley Baptist Medical Center, Harlingen, TX, United States; Department of Neurology, University of Iowa, Iowa City, IA, United States; Stroke Unit, Vall d'Hebron University Hospital, Barcelona, Spain; University Institute of Diagnostic and Interventional Neuroradiology, University of Bern, Inselspital, Bern, Switzerland; University Institute of Diagnostic and Interventional Neuroradiology, University of Bern, Inselspital, Bern, Switzerland; Department of Radiology, Istanbul Aydın University, IAU Stroke Center, Istanbul, Turkey; Department of Radiology, Istanbul Aydın University, IAU Stroke Center, Istanbul, Turkey; Department of Neuroradiology, ASST Grande Ospedale Metropolitano Niguarda, Milan, Italy; Department of Neuroradiology, ASST Grande Ospedale Metropolitano Niguarda, Milan, Italy; Department of Neuroradiology, ASST Grande Ospedale Metropolitano Niguarda, Milan, Italy; Interventional Neuroradiology Unit, Department of Radiology, Hospital Clínico Universitario San Carlos, Madrid, Spain; Interventional Neuroradiology Unit, Department of Radiology, Hospital Clínico Universitario San Carlos, Madrid, Spain; Department of Radiology, Interventional Neuroradiology, Virgen del Rocío University Hospital, Seville, Spain; Neurovascular Laboratory, Instituto de Biomedicina de Sevilla IBiS/University Hospital Virgen del Rocio/CSIC/University of Seville, Sevilla, Spain; Department of Radiology, Interventional Neuroradiology, Virgen del Rocío University Hospital, Seville, Spain; Neuroradiology Unit, A.O. Città della Salute e della Scienza, Turin, Italy; Neuroradiology Unit, A.O. Città della Salute e della Scienza, Turin, Italy; Department of Neurology, Coimbra University, Coimbra, Portugal; Department of Neurology, Coimbra University, Coimbra, Portugal; Department of Neurology, Coimbra University, Coimbra, Portugal; UOSA Neuroradiologia Interventistica, Fondazione Policlinico Universitario A. Gemelli IRCCS, Roma, Italy; UOSA Neuroradiologia Interventistica, Fondazione Policlinico Universitario A. Gemelli IRCCS, Roma, Italy; Diagnostic and Interventional Neuroradiology, Geneva University Hospitals, Geneva, Switzerland; Diagnostic and Interventional Neuroradiology, Geneva University Hospitals, Geneva, Switzerland; Department of Neuroradiology, Henri Mondor Hospital, Creteil, France; Department of Neuroradiology, Henri Mondor Hospital, Creteil, France; Neuroradiology Department, Hospital de Braga, Braga, Portugal; Neuroradiology Department, Hospital de Braga, Braga, Portugal; Interventional Neuroradiology Department, Careggi University Hospital, Florence, Italy; Interventional Neuroradiology Department, Careggi University Hospital, Florence, Italy; Neuroradiology, University Hospital 'San Giovanni di Dio e Ruggi d'Aragona', Salerno, Italy; Department of Radiology, Hospital Universitario 12 de Octubre, Madrid, Spain; Department of Neurology, Hospital Universitario 12 de Octubre, Madrid, Spain; Department of Radiology, Boston Medical Center, Boston University Chobanian and Avedisian School of Medicine, Boston, MA, United States; Department of Neurology, Boston Medical Center, Boston University Chobanian and Avedisian School of Medicine, Boston, MA, United States; Department of Neurology, Boston Medical Center, Boston University Chobanian and Avedisian School of Medicine, Boston, MA, United States; Department of Neuroradiology, Centro Hospitalar Universitário Lisboa Central, Lisboa, Portugal; Department of Neuroradiology, Centro Hospitalar Universitário Lisboa Central, Lisboa, Portugal; Department of Neurology, University of Miami & Jackson Memorial Hospitals, Miami, FL, United States; Department of Neurology, University of Miami & Jackson Memorial Hospitals, Miami, FL, United States; Department of Radiology, Hospital Universitario Central de Asturias, Oviedo, Spain; Department of Radiology, Hospital Universitario Central de Asturias, Oviedo, Spain; Department of Neurology, Eskisehir Osmangazi University School of Medicine, Eskisehir, Turkey; Department of Neurology, Eskisehir Osmangazi University School of Medicine, Eskisehir, Turkey; Department of Interventional Neuroradiology, Rothschild Foundation Hospital, Paris, France; Department of Interventional Neuroradiology, Rothschild Foundation Hospital, Paris, France; Department of Neurology, Cooper Neurological Institute, Camden, NJ, United States; Department of Neurology, Cooper Neurological Institute, Camden, NJ, United States; Department of Human Neurosciences, Interventional Neuroradiology, Policlinico Umberto I, Sapienza University of Rome, Rome, Italy; Department of Human Neurosciences, Interventional Neuroradiology, Policlinico Umberto I, Sapienza University of Rome, Rome, Italy; Department of Neurology, Centro Hospitalar Universitário de São João, Faculty of Medicine University of Porto, Porto, Portugal; Department of Neuroradiology, Centro Hospitalar Universitário São João, Porto, Portugal; Department of Neurosurgery, Jacobs School of Medicine and Biomedical Sciences, University at Buffalo, Buffalo, NY, United States; Department of Neurosurgery, Jacobs School of Medicine and Biomedical Sciences, University at Buffalo, Buffalo, NY, United States; Interventional Neuroradiology, La Paz University Hospital, Hospital La Paz Institute for Health Research-IdiPAZ, Madrid, Spain; Department of Diagnostic and Interventional Neuroradiology, University Hospital Basel, Basel, Switzerland; Department of Diagnostic and Interventional Neuroradiology, University Hospital Basel, Basel, Switzerland; Neuroradiology Unit, Department of Biomedical Sciences and Morphological and Functional Imaging, University of Messina, Messina, Italy; Interventional Neuroradiology, Radiology, Hospital Clínico Universitario Virgen de la Arrixaca, El Palmar, Murcia, Spain; Interventional Neuroradiology, Radiology, Hospital Clínico Universitario Virgen de la Arrixaca, El Palmar, Murcia, Spain; Neuroscience and Stroke Program, Bon Secours Mercy Health St Vincent Hospital, Toledo, OH, United States; Neuroscience and Stroke Program, Bon Secours Mercy Health St Vincent Hospital, Toledo, OH, United States; Stroke Program, Department of Neurology, Hospital Clínico Universitario, Valladolid, Spain; Stroke Program, Department of Neurology, Hospital Clínico Universitario, Valladolid, Spain; Neurology and Stroke Unit, Department of Neuroscience, Bufalini Hospital, Cesena, Italy; Department of Neurology, Hospital de Egas Moniz, Centro Hospitalar Lisboa Ocidental, Lisbon, Portugal; Department of Neurology, The Warren Alpert Medical School of Brown University, Providence, RI, United States; Department of Life Sciences, Health and Health Professions, Endovascular Neurosurgery, Link Campus University, Rome, Italy; Stroke Unit, Vall d'Hebron University Hospital, Barcelona, Spain; Interventional Neuroradiology, Vall d'Hebron University Hospital, Barcelona, Spain; Vall d’Hebron Research Institute, Barcelona, Spain; Interventional Neuroradiology, Vall d'Hebron University Hospital, Barcelona, Spain; Vall d’Hebron Research Institute, Barcelona, Spain

**Keywords:** acute ischaemic stroke, antithrombotics, emergent carotid stenting, mechanical thrombectomy, tandem occlusion

## Abstract

**Introduction:**

Emergent intracranial stenting (EIS) is increasingly employed in the context of the acute ischaemic stroke treatment, but requires intraprocedural antiplatelet therapy (APT), which may raise haemorrhagic risk. This study aimed to evaluate the safety and effectiveness of different APT regimens during EIS.

**Patients and methods:**

This is a subanalysis of the RESISTANT registry, which is a multicenter retrospective registry of patients with acute ischaemic stroke treated with intracranial EIS between 2016 and 2023. Patients receiving intraprocedural antithrombotics were included. Primary efficacy outcomes were stent patency (intraprocedural and within 24 hours) and 3-month mRS. Secondary outcome was successful reperfusion (modified thrombolysis in cerebral infarction ≥ 2b), and the safety outcome was sICH. Multivariable and propensity score-matched analyses were performed.

**Results:**

Among 827 patients, 4 APT strategies were identified: single APT (*n* = 102), oral dual antiplatelet therapy (dAPT) (Aspirin + Clopidogrel or Ticagrelor; *n* = 83), Cangrelor (*n* = 92) and GP IIb/IIIa inhibitors (GPi) (*n* = 550). Intravenous agents (Cangrelor/GPi) showed a trend towards lower risk of intraprocedural stent occlusion compared to oral dAPT (adjusted odds ratio [aOR] 0.30, [95% CI, 0.09–1.01], *P* = .053), though this did not reach statistical significance. GP IIb/IIIa inhibitors continued to demonstrate a protective trend at 24 hours (aOR 0.25, [95% CI, 0.06–0.99], *P* = .047), without a significant increase in sICH. Both intravenous agents were independently associated with higher odds of successful final reperfusion (odds ratio [OR] 4.35, [95% CI, 1.57–12.09], *P* = .001). No significant differences emerged between GPi and Cangrelor in matched analysis. No significant difference was observed on good functional outcome between APT strategies.

**Conclusion:**

In the setting of EIS, intravenous APT agents (Cangrelor or GPi) were associated with improved stent patency and higher rates of successful reperfusion, without a significant increase in symptomatic haemorrhage.

## Graphical Abstract

**Graphical abstract:**
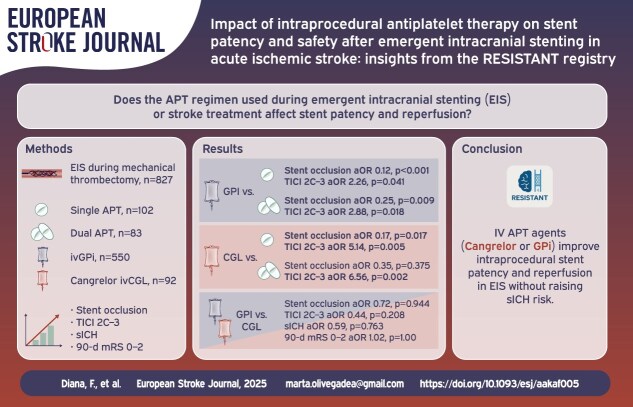

## Introduction

Emergent intracranial stenting (EIS) has become a critical strategy in acute ischaemic stroke (AIS) patients with LVO, as a first-line or rescue strategy when an intracranial atherosclerotic stenosis (ICAS) is suspected, or when mechanical thrombectomy (MT) fails to achieve adequate reperfusion. Growing evidence from observational studies suggests that EIS may improve outcomes in selected patients, both in anterior and posterior circulation strokes, without significantly increasing the risk of haemorrhagic complications.^1–6^

Despite its increasing adoption, EIS remains off-label, and its procedural success hinges on maintaining acute stent patency—a challenge particularly relevant in the thrombogenic environment of acute stroke. To prevent early in-stent thrombosis, intraprocedural antiplatelet therapy (APT) is commonly administered,^7^ even though there is still a concern regarding the risk of haemorrhagic transformation (HT) that it may carry. Balancing these competing risks is especially challenging given the lack of standardised protocols and the wide heterogeneity in agent selection, timing and dosing across centers.

To date, there is no consensus on the optimal intraprocedural APT strategy for patients undergoing EIS. While some centers favor oral dual antiplatelet therapy (dAPT) with Aspirin and P2Y12 inhibitors, others use intravenous agents such as Cangrelor or GP IIb/IIIa inhibitors (GPi) due to their rapid onset and short half-lives. However, a head-to-head comparison across APT strategies is lacking, and the evidence base remains limited to small, single-center studies.

To address these gaps, we aimed to evaluate the stent patency and risk of sICH of different intraprocedural antithrombotic strategies during acute stroke treatment with intracranial EIS for refractory LVO, using data from a large, international, multicenter registry.

## Patients and methods

### Study design and population

We conducted a retrospective analysis of the RESISTANT (Registry of Endovascular Salvage for Intracranial Stenting in Thrombectomy-refractory Stroke) registry, a multicenter, international retrospective observational cohort study. This registry includes consecutive adult patients with AIS who underwent intracranial EIS following failed MT at 36 comprehensive stroke centers (Spain, Italy, Portugal, France, Switzerland, Turkey, United States), treated between 1 January 2016 and 30 July 2023. Patients were selected for endovascular treatment (EVT) according to local institutional protocols. Inclusion criteria for this analysis were: (1) age ≥ 18 years, (2) rescue stenting performed in the anterior or posterior circulation, (3) any antiplatelet treatment administered during the procedure and (4) a postoperative antiplatelet regimen initiated after 24 hours. Patients who did not receive antiplatelet treatment or did not undergo stenting were excluded. Procedures and follow-up were conducted in accordance with standard-of-care practices.

This study adheres to the Strengthening the Reporting of Observational Studies in Epidemiology (STROBE) guidelines. Ethical approval was granted by the coordinating center’s ethics committee, which waived the need for informed consent due to the retrospective nature of the study.

### Clinical variables and Intraprocedural antiplatelet regimens

Baseline demographic, clinical, radiological and procedural characteristics were collected. EVT was performed according to local institutional protocols. Data included stroke severity (NIHSS), baseline imaging (ASPECTS), occlusion location, technique used (stent retriever, aspiration or combined), anesthesia type, number of thrombectomy passes and time from stroke onset to reperfusion. In patients undergoing double antiplatelet therapy for an already known intracranial stenosis the acute ischaemic stroke was considered as a failure of the best medical therapy.

Four intraprocedural antiplatelet strategies (Table S1) were identified: single antiplatelet therapy (sAPT), consisting of Aspirin alone (administered orally, intravenously or rectally at doses ranging from 100 to 500 mg); dAPT, defined as Aspirin combined with a P2Y12 inhibitor (either Clopidogrel 300 mg or Ticagrelor 180 mg); intravenous Cangrelor, delivered as a weight-based bolus and infusion with a duration determined by institutional protocols; and GPi, with Tirofiban, Eptifibatide or Abciximab, also administered as a weight-based bolus plus infusion. Antiplatelet agents administered solely in response to intraprocedural thrombosis were not considered part of the initial treatment strategy.

### Outcomes

The primary effectiveness outcome was the rate of intraoperative stent occlusion, defined as a complete occlusion of the deployed stent. Secondary efficacy outcomes included stent patency within 24 hours post-procedure (assessed in any patient with CT angiography, MR angiography, digital subtraction angiography or transcranial Doppler), reperfusion status at the end of the procedure (measured using the modified thrombolysis in cerebral infarction [mTICI] scale),^8^ functional outcomes, measured by the mRS at 90 days. mRS at 90 days was assessed by vascular neurologists during routine follow-up visits or by certified study nurses via standardised telephone interviews. Successful recanalisation was defined as mTICI 2b-3 and complete recanalisation as mTICI 2c-3. Imaging evaluations, including mTICI scoring, were adjudicated by local interventionists.

Safety outcomes included HT at day 1, parenchymal haemorrhage (PH1 or PH2) according to the ECASS II trial,^9^ and sICH, defined as any intracranial haemorrhage on post-treatment imaging within 7 days, associated with a ≥ 4-point deterioration on the NIHSS score or death.

### Statistical analysis

Continuous variables were summarised as medians with interquartile ranges, and categorical variables as counts with percentages. Baseline and procedural characteristics were compared using χ^2^ tests for categorical variables and ANOVA or Kruskal–Wallis tests for continuous variables, as appropriate. Univariable and multivariable logistic regression analyses were used to assess associations between intraprocedural antiplatelet therapy and binary clinical or radiological outcomes: intraprocedural stent occlusion, 24-hour stent occlusion, complete reperfusion (mTICI > 2c), sICH and 90-day functional independence (mRS 0–2). Covariates included age, sex, pre-stroke mRS, baseline NIHSS, IV thrombolysis, ASPECTS, onset-to-recanalisation time, occlusion site, vascular territory, tandem occlusion and pre-stenting recanalisation status. Missing data were not imputed for descriptive analyses and unmatched regression models, which were conducted on available cases only.

Pairwise comparisons were pre-planned between treatment groups (dAPT vs sAPT, Cangrelor vs sAPT, GPi vs sAPT, Cangrelor vs dAPT, GPi vs dAPT, intravenous agents vs oral dAPT, GPi vs Cangrelor) to test predefined hypotheses and avoid type I error inflation. To further evaluate Cangrelor vs GPi-based strategies, a propensity score-matched analysis was performed using logistic regression-derived scores from the covariates. Missing data were imputed using multiple imputation by chained equations (MICE; mice package, R). Matching was conducted using nearest-neighbor methods, and balance was assessed via standardised mean differences (SMDs). Additional regression adjustment was performed if post-matching imbalances (SMD ≥ 0.1) were identified. Statistical significance was set at *P* < .05. Analyses were conducted using R (version 4.3.0; R Foundation for Statistical Computing, Vienna, Austria).

## Results

### Population

**Figure 1:**
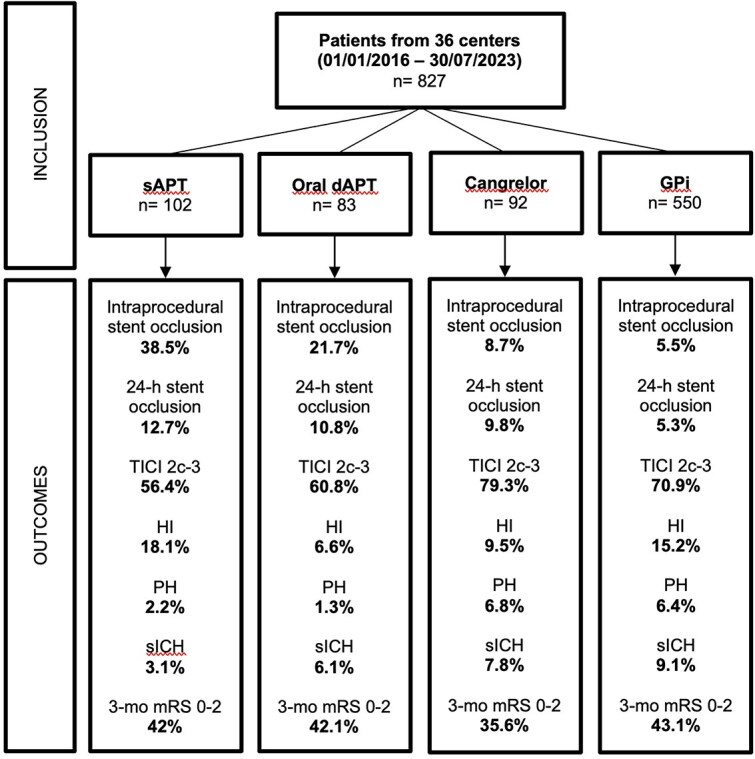
Flow diagram of patients included in the study.

A total of 827 patients met inclusion criteria (Figure 1). Four intraprocedural antiplatelet strategies were identified: sAPT with Aspirin alone (*n* = 102), oral dAPT (Aspirin + Clopidogrel or Ticagrelor; *n* = 83), IV Cangrelor (*n* = 92) and GPi (*n* = 550). At baseline, 22% of patients were on Aspirin and 6.4% on dAPT. The dAPT group had the highest rate of pretreatment with dAPT (23.2%). Oral anticoagulation use (15.4%) and IV thrombolysis administration (23%) were similar across groups. A total of 592 patients (71.9%) presented with anterior circulation occlusion, and 231 of them (28.1%) presented with posterior circulation occlusion, without differences between groups. Intracranial stenting was performed without previous MT in 12.5% of cases, as rescue strategy after failed MT in 85.7% of cases. Baseline characteristics are summarised in Table 1 and outcomes in Table S2.

**Table 1:** Baseline characteristics according to intraprocedural antithrombotic regimen.

	**SAPT-ASA (*n* = 102)**	**Oral DAPT (*n* = 83)**	**Cangrelor (*n* = 92)**	**GPi (*n* = 550)**	**Total (*n* = 827)**	***P*-value**
Age, median (IQR)	66.0 (56.2, 78.8)	65.0 (59.0, 73.0)	67.0 (57.0, 79.2)	68.0 (59.0, 77.0)	67.0 (59.0, 77.0)	.400
Sex, F, *n*, %	31 (30.4%)	25 (30.1%)	35 (38.5%)	203 (36.9%)	294 (35.6%)	.387
Baseline mRS, median (IQR)	0.0 (0.0, 1.0)	0.0 (0.0, 1.0)	0.0 (0.0, 0.0)	0.0 (0.0, 1.0)	0.0 (0.0, 1.0)	.074
HBP	71 (71.0%)	67 (80.7%)	59 (67.8%)	404 (73.5%)	601 (73.3%)	.267
DLP	38 (38.4%)	50 (60.2%)	26 (29.9%)	259 (47.1%)	373 (45.5%)	<.001
DM	34 (34.3%)	29 (34.9%)	33 (38.4%)	197 (35.8%)	293 (35.8%)	.947
AF	17 (17.2%)	8 (9.6%)	15 (17.4%)	77 (14.0%)	117 (14.3%)	.412
CAD	16 (16.2%)	14 (16.9%)	14 (16.3%)	63 (11.5%)	107 (13.1%)	.274
CRF	28 (28.0%)	15 (18.1%)	15 (17.4%)	92 (16.7%)	150 (18.3%)	.065
Smokers	43 (43.9%)	36 (43.4%)	27 (34.6%)	232 (42.2%)	338 (41.8%)	.584
Past stroke	30 (30.3%)	30 (36.1%)	23 (26.7%)	138 (25.1%)	221 (27.0%)	.166
**Previous APT**						<.001
SAPT	19 (19.4%)	18 (22.0%)	23 (27.7%)	118 (21.6%)	178 (22.0%)	
DAPT	6 (6.1%)	19 (23.2%)	4 (4.8%)	23 (4.2%)	52 (6.4%)	
Previous OAC	11 (11.1%)	10 (12.0%)	17 (20.0%)	88 (16.0%)	126 (15.4%)	.302
Known ICAD	9 (9.0%)	15 (18.1%)	12 (13.8%)	25 (4.5%)	61 (7.4%)	<.001
Baseline NIHSS, median (IQR)	14.5 (9.0, 20.0)	10.0 (6.0, 17.0)	13.0 (7.0, 19.0)	13.0 (8.0, 19.0)	13.0 (8.0, 19.0)	.094
**Occlusion side**						.111
Right	35 (34.3%)	21 (25.3%)	29 (31.5%)	187 (34.1%)	272 (33.0%)	
Left	47 (46.1%)	34 (41.0%)	33 (35.9%)	237 (43.2%)	351 (42.5%)	
Posterior	20 (19.6%)	28 (33.7%)	30 (32.6%)	124 (22.6%)	202 (24.5%)	
**Occlusion location**						.516
ICA-T	21 (20.6%)	12 (14.5%)	13 (14.1%)	95 (17.3%)	141 (17.1%)	
M1	52 (51.0%)	34 (41.0%)	33 (35.9%)	259 (47.2%)	378 (45.8%)	
M2	5 (4.9%)	5 (6.0%)	10 (10.9%)	40 (7.3%)	60 (7.3%)	
M3/M4	0 (0.0%)	0 (0.0%)	0 (0.0%)	3 (0.5%)	3 (0.4%)	
A1/A2	1 (1.0%)	0 (0.0%)	1 (1.1%)	7 (1.3%)	9 (1.1%)	
A3/A4	0 (0.0%)	0 (0.0%)	0 (0.0%)	1 (0.2%)	1 (0.1%)	
V4	5 (4.9%)	8 (9.6%)	8 (8.7%)	40 (7.3%)	61 (7.4%)	
BA proximal	13 (12.7%)	11 (13.3%)	17 (18.5%)	56 (10.2%)	97 (11.7%)	
BA middle	3 (2.9%)	7 (8.4%)	8 (8.7%)	24 (4.4%)	42 (5.1%)	
BA distal	1 (1.0%)	2 (2.4%)	0 (0.0%)	8 (1.5%)	11 (1.3%)	
P1	1 (1.0%)	2 (2.4%)	2 (2.2%)	13 (2.4%)	18 (2.2%)	
P2	0 (0.0%)	1 (1.2%)	0 (0.0%)	1 (0.2%)	2 (0.2%)	
**Circulation**						.011
Anterior	79 (77.5%)	51 (62.2%)	57 (62.0%)	405 (74.0%)	592 (71.9%)	
Posterior	23 (22.5%)	31 (37.8%)	35 (38.0%)	142 (26.0%)	231 (28.1%)	
ASPECTS, median (IQR)	9.0 (8.0, 10.0)	9.0 (8.0, 10.0)	9.0 (8.0, 10.0)	9.0 (8.0, 10.0)	9.0 (8.0, 10.0)	.054
**ICAS degree**						<.001
<25%	0 (0.0%)	1 (1.2%)	1 (1.1%)	9 (1.8%)	11 (1.4%)	
25%–49%	1 (1.0%)	0 (0.0%)	3 (3.3%)	5 (1.0%)	9 (1.2%)	
50%–75%	1 (1.0%)	7 (8.4%)	9 (9.8%)	18 (3.6%)	35 (4.5%)	
>75%	22 (21.6%)	38 (45.8%)	30 (32.6%)	125 (24.9%)	215 (27.6%)	
100%	78 (76.5%)	37 (44.6%)	49 (53.3%)	346 (68.8%)	510 (65.4%)	
**Mori classification**						.372
A	40 (41.7%)	25 (32.5%)	33 (36.7%)	140 (31.1%)	238 (33.4%)	
B	35 (36.5%)	32 (41.6%)	37 (41.1%)	175 (38.9%)	279 (39.1%)	
C	21 (21.9%)	20 (26.0%)	20 (22.2%)	135 (30.0%)	196 (27.5%)	
Onset-recanalisation, median (IQR)	401.5 (248.2, 701.7)	439.0 (281.5, 902.0)	353.0 (266.0, 492.5)	387.0 (249.5, 610.2)	390.0 (253.5, 643.0)	.170
IVT	28 (27.5%)	16 (19.3%)	23 (25.0%)	123 (22.4%)	190 (23.0%)	.550
**Stenting strategy**						.008
1st line	10 (9.8%)	20 (24.1%)	12 (13.0%)	61 (11.1%)	103 (12.5%)	
Rescue	92 (90.2%)	63 (75.9%)	80 (87.0%)	488 (88.9%)	723 (87.5%)	
Number of passes present, median (IQR)	2.0 (1.0, 3.0)	2.0 (1.0, 3.0)	2.0 (1.0, 3.0)	2.0 (1.0, 3.0)	2.0 (1.0, 3.0)	.475
**MT technique**						<.001
Stent retriever only	18 (19.8%)	9 (16.1%)	2 (2.6%)	90 (18.8%)	119 (16.9%)	
Aspiration only	10 (11.0%)	16 (28.6%)	34 (43.6%)	98 (20.5%)	158 (22.5%)	
Combined	63 (69.2%)	31 (55.4%)	42 (53.8%)	290 (60.7%)	426 (60.6%)	
**TICI pre-stenting**						.010
TICI 0	27 (29.0%)	17 (23.9%)	25 (32.1%)	163 (31.9%)	232 (30.8%)	
TICI 1	7 (7.5%)	6 (8.5%)	17 (21.8%)	68 (13.3%)	98 (13.0%)	
TICI 2a	20 (21.5%)	10 (14.1%)	11 (14.1%)	78 (15.3%)	119 (15.8%)	
TICI 2b	22 (23.7%)	14 (19.7%)	7 (9.0%)	66 (12.9%)	109 (14.5%)	
TICI 2c	7 (7.5%)	4 (5.6%)	1 (1.3%)	28 (5.5%)	40 (5.3%)	
TICI 3	10 (10.8%)	20 (28.2%)	17 (21.8%)	108 (21.1%)	155 (20.6%)	
**Pre-stenting TICI**						.055
TICI 0-2a	54 (58.1%)	33 (46.5%)	53 (67.9%)	309 (60.5%)	449 (59.6%)	
TICI 2b-3	39 (41.9%)	38 (53.5%)	25 (32.1%)	202 (39.5%)	304 (40.4%)	
**Final TICI**						.002
TICI 0	11 (10.9%)	4 (5.1%)	1 (1.1%)	15 (2.7%)	31 (3.8%)	
TICI 1	0 (0.0%)	0 (0.0%)	1 (1.1%)	4 (0.7%)	5 (0.6%)	
TICI 2a	7 (6.9%)	8 (10.1%)	0 (0.0%)	30 (5.5%)	45 (5.5%)	
TICI 2b	26 (25.7%)	19 (24.1%)	17 (18.5%)	111 (20.2%)	173 (21.1%)	
TICI 2c	12 (11.9%)	6 (7.6%)	12 (13.0%)	72 (13.1%)	102 (12.4%)	
TICI 3	45 (44.6%)	42 (53.2%)	61 (66.3%)	317 (57.7%)	465 (56.6%)	
**Anesthesia type**						.007
General	69 (67.6%)	36 (43.4%)	43 (46.7%)	275 (50.0%)	423 (51.1%)	
Sedation	30 (29.4%)	45 (54.2%)	49 (53.3%)	264 (48.0%)	388 (46.9%)	
Local	3 (2.9%)	2 (2.4%)	0 (0.0%)	11 (2.0%)	16 (1.9%)	
Angioplasty pre-stenting	56 (55.4%)	39 (47.0%)	28 (30.4%)	299 (54.4%)	422 (51.1%)	<.001
Angioplasty post-stenting	30 (30.0%)	20 (24.1%)	40 (43.5%)	64 (11.7%)	154 (18.8%)	<.001
**Stent type**						<.001
Balloon-expandable	16 (16.2%)	26 (31.3%)	30 (32.6%)	88 (16.0%)	160 (19.4%)	
Self-expandable	83 (83.8%)	57 (68.7%)	62 (67.4%)	462 (84.0%)	664 (80.6%)	
**Number of stents**						.163
0	1 (1.0%)	0 (0.0%)	0 (0.0%)	0 (0.0%)	1 (0.1%)	
1	83 (84.7%)	77 (92.8%)	80 (87.0%)	504 (92.0%)	744 (90.6%)	
2	14 (14.3%)	5 (6.0%)	11 (12.0%)	40 (7.3%)	70 (8.5%)	
3	0 (0.0%)	1 (1.2%)	1 (1.1%)	3 (0.5%)	5 (0.6%)	
4	0 (0.0%)	0 (0.0%)	0 (0.0%)	1 (0.2%)	1 (0.1%)	
**Intraoperative heparin**						<.001
Lines/saline	3 (3.3%)	5 (6.5%)	0 (0.0%)	25 (5.0%)	33 (4.6%)	
Yes	20 (21.7%)	45 (58.4%)	3 (5.7%)	68 (13.7%)	136 (18.9%)	
APT switch time						<.001
Immediate	9 (13.4%)	1 (11.1%)	18 (20.2%)	3 (0.7%)	31 (5.2%)	
Within 24 h	53 (79.1%)	7 (77.8%)	41 (46.1%)	353 (81.5%)	454 (75.9%)	
After 24 h	5 (7.5%)	1 (11.1%)	30 (33.7%)	77 (17.8%)	113 (18.9%)	
**Statin therapy**						<.001
No	15 (23.1%)	8 (11.8%)	32 (51.6%)	83 (24.1%)	138 (25.6%)	
Low intensity	8 (12.3%)	1 (1.5%)	21 (33.9%)	28 (8.1%)	58 (10.7%)	
High intensity	42 (64.6%)	59 (86.8%)	9 (14.5%)	234 (67.8%)	344 (63.7%)	

Abbreviations: AF = atrial fibrillation; APT = antiplatelet therapy; ASA = acetylsalicylic acid; ASPECTS = Alberta Stroke Program Early CT Score; CRF = cardiovascular risk factors; dAPT = dual antiplatelet therapy; DLP = dyslipidemia; DM = diabetes mellitus; GPi = GP IIb/IIIa inhibitors; HBP = high blood pressure; ICAS = intracranial atherosclerotic stenosis; ICAD = intracranial artery disease; IVT = intravenous thrombolysis; MT = mechanical thrombectomy; OAC = oral anticoagulation; sAPT = single antiplatelet therapy; TICI = thrombolysis in cerebral infarction.

### Intraprocedural stent occlusion

The effect of the APT regimens on occlusion at different time points is summarised in Table 2. Intraprocedural stent occlusion occurred in 93/827 (11.3%) of cases and was associated with a lower rate of good functional outcome (mRS 0–2 33.3% vs 42.2%), and higher rate of sICH (12.2% vs 7.9%; Table S3). In multivariable analysis, both Cangrelor and GPi were associated with reduced risk of intraprocedural occlusion compared to sAPT (Cangrelor: odds ratio [OR] 0.17, [95% CI, 0.04–0.79], *P* = .017; GPi: OR 0.12, [95% CI, 0.05–0.30], *P* < .001; Table S4). IV agents were also associated with lower risk compared to oral dAPT (OR 0.30, [95% CI, 0.09–1.01], *P* = .053).

**Table 2:** Outcomes of different groups.

	**Intraprocedural occlusion**		**Occlusion within 24 h**		**TICI 2c-3**		**HT (HI1-PH2)**		**sICH**		**mRS**	
	**aOR (95% CI)**	***P*-value**	**aOR (95% CI)**	***P*-value**	**aOR (95% CI)**	***P*-value**	**aOR (95% CI)**	***P*-value**	**aOR (95% CI)**	***P*-value**	**aOR (95% CI)**	***P*-value**
Oral dAPT vs sAPT	0.48 (0.15–1.57)	.380	4.94 (0.71–34.18)	.146	0.78 (0.26–2.37)	.941	0.22 (0.04–1.08)	.068	2.09 (0.18–23.76)	.859	0.66 (0.20–2.19)	.806
Cangrelor vs sAPT	0.17 (0.04–0.79)	.017	2.13 (0.26–17.58)	.790	5.14 (1.45–18.28)	.005	0.71 (0.20–2.48)	.895	4.99 (0.52–47.73)	.254	0.71 (0.21–2.48)	.896
GPi vs sAPT	0.12 (0.05–0.30)	<.001	1.23 (0.23–6.54)	.989	2.26 (1.03–4.97)	.041	0.66 (0.29–1.48)	.548	2.92 (0.43–19.97)	.471	0.73 (0.31–1.71)	.775
IV APT vs oral dAPT	0.30 (0.09–1.01)	.053	0.33 (0.08–1.36)	.181	4.35 (1.57–12.09)	.001	3.02 (0.68–13.41)	.218	1.82 (0.32–10.33)	.803	1.09 (0.38–3.10)	.996
GPi vs Cangrelor	0.72 (0.16–3.30)	.944	0.58 (0.12–2.84)	.808	0.44 (0.15–1.30)	.208	0.92 (0.30–2.76)	.997	0.59 (0.14–2.44)	.763	1.02 (0.36–2.87)	1.000

Abbreviations: aOR = adjusted odds ratio; APT = antiplatelet therapy; dAPT = dual antiplatelet therapy; GPi = GP IIb/IIIa inhibitors; HT = haemorrhagic transformation; sAPT = single antiplatelet therapy; TICI = thrombolysis in cerebral infarction.

Treatment of the intraprocedural occlusion was done in 77 cases (14.6%), mainly in the sAPT and dAPT groups. The administration of IV drugs in 56 patients (GPi in 51 cases and Cangrelor in 5 cases) was effective in 42 of them. Mechanical strategies, including angioplasty or MT tried in 21 patients were effective in 13 patients.

### Reperfusion, stent occlusion after 24 hours and functional outcomes

Both Cangrelor and GPi were associated with higher odds of successful reperfusion versus sAPT (Cangrelor: OR 5.14, [95% CI, 1.45–18.28], *P* = .005; GPi: OR 2.26, [95% CI, 1.03–4.97], *P* = .041) and versus dAPT (IV APT vs oral dAPT: OR 4.35 [95% CI, 1.57–12.09], *P* = .001; Table 1 and Table S5).

Within 24 hours, stent occlusion occurred in 60/827 (7.3%) of cases. GP IIb/IIIa inhibitors use remained significantly associated with reduced occlusion risk compared to oral dAPT (OR 0.25, [95% CI, 0.06–0.99], *P* = .047; Table S6), although Cangrelor did not reach statistical significance.

There were no significant differences in good functional outcomes (mRS 0–2) at 90 days between groups (overall: 42.2%). After adjustment, no intraprocedural antiplatelet strategy was independently associated with better outcomes (Table S7).

### Safety outcomes

Haemorrhagic transformation occurred in 19.7% of patients, and sICH occurred in 7.9%. In the univariate analysis, GPi was associated with a statistically significant higher HT compared to oral dAPT (OR 3.15, [95% CI, 1.03–9.61], *P* = .041), but it was not confirmed after adjusting for confounders (OR 2.91, [95% CI, 0.69–12.25], *P* = .217). Intravenous agents compared with oral dAPT showed a trend towards a higher HT (OR 3.02, [95% CI, 0.68–13.41], *P* = .218), without difference between them (GPi vs Cangrelor: OR 0.92, [95% CI, 0.30–2.76], *P* = .997). No significant differences in sICH rates were observed between groups before and after adjusting for confounders (Tables S2 and S8).

### Propensity score analysis

We performed a propensity score-matched analysis to compare Cangrelor and GPi-based therapies. After matching Cangrelor and GPi groups, covariate balance was acceptable (Table S9). Logistic regression showed no significant differences in intraprocedural or 24-hour stent occlusion, reperfusion success, sICH or 90-day mRS between the 2 groups (Table 3).

**Table 3:** Propensity score-matched (PSM) analysis to compare Cangrelor and GPi-based therapies.

**PSM analysis Cangrelor vs GPi**		
	**OR (95% CI)**	***P*-value**
Intraprocedural stent occlusion	1.76 (0.43–7.17)	.425
Occlusion stent within 24 h	2.20 (0.49–9.90)	.299
sICH	0.91 (0.23–3.57)	.897

Abbreviations: OR = odds ratio; GPi = GP IIb/IIIa inhibitors.

## Discussion

In this large multicenter cohort of patients undergoing EIS for acute ischaemic stroke, we aimed to assess the effectiveness and safety of commonly used intraprocedural APT strategies. We report 3 key findings: (1) Cangrelor and GPi were associated with a lower risk of stent occlusion during the procedure and within 24 hours, compared to sAPT; (2) both IV agents were linked to higher odds of successful recanalisation without compromising safety; (3) neither intravenous nor sAPT strategies were associated with significant differences in functional outcomes or sICH.

These results provide important real-world evidence to guide APT selection during EIS, a practice that remains off-label and highly heterogeneous across institutions. Several retrospective studies have shown that EIS, either as a rescue strategy for failed MT^1–6^ or as a first-line intervention in patients with underlying ICAS,^10^ is associated with better functional outcomes without a significantly increased risk of sICH, compared to leaving the persistent occlusion untreated. However, the evidence regarding the management of antiplatelet therapy in such cases is scarce and is based on small cohorts and lack of comparisons. Baek et al.^11^ demonstrated that, in acute stroke caused by ICAS-related LVO (ICAS-LVO), the combination of EIS and GPi infusion may represent an optimal rescue approach when frontline MT fails. In their cohort, rescue stenting with GPi infusion was associated with a high recanalisation rate (TICI 2b-3 in 98.0%) and excellent stent patency on follow-up (98.0%, *P* < .001). Our findings are consistent with these results. In our cohort of 550 GPi-treated patients, 91.1% achieved a TICI 2b-3 and 24-hour stent patency was observed in 94.7% of cases, with a sICH rate of 9.1%. Garayzade et al. similarly reported no excess risk of sICH with GPi use.^12^ Our analysis further expands on these findings by offering the largest comparative dataset to date and including Cangrelor, a P2Y12 inhibitor with pharmacokinetic properties particularly appealing for acute stroke care due to its short half-life and intravenous delivery.^12^ Moreover, we provided additional insight into the haemorrhagic risk by analysing all types of haemorrhagic transformation (HT). Although there was a trend towards a higher risk of HT with intravenous agents (both GPi and Cangrelor), this was not statistically significant. Our propensity-matched analysis showed no significant differences between Cangrelor and GPi across all efficacy and safety outcomes, suggesting that either agent may be acceptable for use in EIS. These findings are consistent with previous cervical tandem occlusion studies in which both agents were used.^13^^,^^14^ This finding is clinically relevant, especially in settings where access, cost or contraindications may influence agent selection.

Maintaining stent patency during and after the procedure emerges as a crucial determinant of outcome in patients undergoing EIS. In our cohort, intraprocedural stent occlusion occurred in over 11% of cases and was associated with a clear trend towards worse functional outcomes (33.3% vs 42.2%), and higher rates of sICH (12.2% vs 7.9%). No significant difference was observed in good functional outcomes between antiplatelet therapies, suggesting that stent patency alone may not be the sole determinant of clinical success. Functional outcome is likely influenced by additional factors occurring after treatment, such as in-hospital and post-admission complications, patients’ comorbidities and the post-procedural antiplatelet regimen needed to maintain the long-term patency of the stent. Notably in our cohort, GPi was frequently employed as a rescue strategy in cases where initial APT had failed, proving effective in many cases (82% of rescues). This GPi use highlights its potency and raises the question of whether earlier administration—rather than rescue use—might prevent occlusions and obviate the need for additional interventions, that might potentially increase the haemorrhagic risk related to the amount of administrated APT drugs. This finding is clinically relevant, especially in settings where access, cost or contraindications may influence agent selection.

Finally, the complexity of antithrombotic management during EIS is a critical consideration, as antiplatelet use for rescue stenting during MT has not been evaluated in randomised controlled trials, and clinical practice currently relies on operator consensus. Patients often receive a combination of thrombolysis, anticoagulation and 1 or more antiplatelet agents concurrently, many of whom may have been on preoperative antiplatelets or anticoagulants. This multifaceted approach introduces significant variability and potential interactions that complicate clinical decision-making. Our study provides valuable observational data to inform these decisions, however, the concurrent use of multiple antithrombotic therapies underscores the need for further research to optimise treatment protocols and balance efficacy against haemorrhagic risks.

## Limitations

This study has several limitations inherent to its retrospective, observational design. First, treatment allocation was not randomised and reflected center-specific protocols, introducing potential selection bias. Although we performed multivariable adjustments and propensity score matching, residual confounding cannot be excluded. Second, Cangrelor use was limited to a small subset of participating centers (7 of 36), and some antiplatelet strategies were underrepresented in individual sites, precluding center-level clustering adjustment. Third, dosing regimens, infusion durations and timing of APT administration varied across centers and were not standardised, which may affect both efficacy and safety outcomes. We did not adjust for center-level clustering in the analysis due to the highly unbalanced distribution of treatments across centers. Fourth, we were unable to assess the impact of heparin therapy due to the low number of patients receiving heparin in saline solution, the lack of standardisation in its administration and the variability in achieving a standard dose. However, the low use of heparin may reflect the clinical practices of the included centers, and its use may be more prevalent in other institutions. Fifth, in our cohort, the rate of IVT was low, and we cannot rule out that IVT may influence the decision to perform an EIS. Sixth, the decision on imaging assessments for stent patency was performed locally and was not centrally adjudicated. Finally, while our findings suggest potential benefits of intravenous agents, the study was not powered to detect differences in long-term clinical outcomes, and unmeasured factors may have influenced functional recovery.

## Conclusion

In this large, multicenter analysis of patients undergoing EIS, the use of intravenous antiplatelet agents—Cangrelor or GP IIb/IIIa inhibitors—was associated with improved stent patency and higher rates of successful reperfusion, without a significant increase in symptomatic intracranial haemorrhage. These findings support the potential role of intravenous agents in optimising intraprocedural antithrombotic management during acute intracranial stenting. Further prospective, controlled studies are needed to validate these results and establish evidence-based protocols for antiplatelet use in the setting of rescue stenting.

## Supplementary Material

aakaf005_RESISTANTAPT_Supplementary
