# Supplementary material for: Impact of intraprocedural antiplatelet therapy on stent patency and safety after emergent intracranial stenting in acute ischaemic stroke: insights from the RESISTANT registry
Source: Eur Stroke J. 2026 Jan 1;11(1):aakaf005. doi: 10.1093/esj/aakaf005 (PMC12964110; doi:10.1093/esj/aakaf005)
Supplement: aakaf005_RESISTANTAPT_Supplementary [file aakaf005_resistantapt_supplementary.docx]

**Impact of Intraprocedural Antiplatelet Therapy on Stent Patency and Safety after Emergent Intracranial Stenting in Acute Ischemic Stroke: Insights from the RESISTANT Registry**

**Supplementary Table 1.** Intraprocedural antiplatelets used during treatment.

**Supplementary Table 2.** Outomes according to intraprocedural antithrombotic regimen.

**Supplementary Table 3.** Outcomes according to intraprocedural stent occlusion.

**Supplementary Table 4.** Univariable and multivariable logistic regression analysis for intraprocedural stent occlusion.

**Supplementary Table 5.** Univariable and multivariable logistic regression analysis for TICI 2c-3.

**Supplementary Table 6.** Univariable and multivariable logistic regression analysis for occlusion within 24 hours.

**Supplementary Table 7.** Univariable and multivariable logistic regression analysis for 3 months mRS.

**Supplementary Table 8.** Univariable and multivariable logistic regression analysis for sICH.

**Supplementary Table 9.** Standard mean difference after IPTW to compare GPi and Cangrelor.

**Supplementary Table 1.** Intraprocedural antiplatelets used during treatment.

| **Group** | **Type** | **Dosage** | **Number of patients** |
| --- | --- | --- | --- |
| **ASA** | Oral | 100-600 mg | 5 (4.9%) |
|  | Intravenous | 250-1000 mg | 93 (91.1%) |
|  | Rectal | 300 mg | 4 (4%) |
| **Oral DAPT** | with Clopidogrel | 300 mg | 43 (51.8%) |
|  | with Ticagrelor | 180 mg | 40 (49.2%) |
| **Cangrelor** | Alone | 30 µg/kg + 4 µg/kg/min | 71 (77.2%) |
|  | with ASA | 30 µg/kg + 4 µg/kg/min | 21 (22.8%) |
| **GPi** | Tirofiban | 25 µg/kg + 0.1 µg/kg | 228 (41.4%) |
|  | Tirofiban + ASA | 25 µg/kg + 0.1 µg/kg | 188 (34.1%) |
|  | Eptifibatide | 45-90 µg/kg + 0.5-1 µg/kg | 27 (5%) |
|  | Eptifibatide + ASA | 45-90 µg/kg + 0.5-1 µg/kg | 93 (17%) |
|  | Abciximab | 0.25 mg/kg bolus + 0.125 µg/kg/min | 6 (1.1%) |
|  | Abciximab + ASA | 0.25 mg/kg bolus + 0.125 µg/kg/min | 8 (1.4%) |

**Supplementary Table 2.** Outcomes according to intraprocedural antithrombotic regimen.

|  | **SAPT-ASA (N=102)** | **Oral DAPT (N=83)** | **Cangrelor (N=92)** | **GPi (N=550)** | **Total (N=827)** | **p value** |
| --- | --- | --- | --- | --- | --- | --- |
| **Final TICI** |  | | | | | 0.002 |
| TICI 0 | 11 (10.9%) | 4 (5.1%) | 1 (1.1%) | 15 (2.7%) | 31 (3.8%) |  |
| TICI 1 | 0 (0.0%) | 0 (0.0%) | 1 (1.1%) | 4 (0.7%) | 5 (0.6%) |  |
| TICI 2a | 7 (6.9%) | 8 (10.1%) | 0 (0.0%) | 30 (5.5%) | 45 (5.5%) |  |
| TICI 2b | 26 (25.7%) | 19 (24.1%) | 17 (18.5%) | 111 (20.2%) | 173 (21.1%) |  |
| TICI 2c | 12 (11.9%) | 6 (7.6%) | 12 (13.0%) | 72 (13.1%) | 102 (12.4%) |  |
| TICI 3 | 45 (44.6%) | 42 (53.2%) | 61 (66.3%) | 317 (57.7%) | 465 (56.6%) |  |
| **Successful recanalization** |  | | | | | |
| TICI 2b-3 | 83 (82.2%) | 67 (84.8%) | 90 (97.8%) | 500 (91.1%) | 740 (90.1%) | < 0.001 |
| TICI 2c-3 | 57 (56.4%) | 48 (60.8%) | 73 (79.3%) | 389 (70.9%) | 567 (69.1%) | 0.001 |
| **Intraprocedural stent occlusion** | 37 (38.5%) | 18 (21.7%) | 8 (8.7%) | 30 (5.5%) | 93 (11.3%) | < 0.001 |
| **Treatment of intraprocedural occlusion** | 32 (43.2%) | 15 (24.2%) | 6 (9.0%) | 24 (7.4%) | 77 (14.6%) | < 0.001 |
| **Residual ICAS** | 49 (48.0%) | 52 (62.7%) | 49 (54.4%) | 291 (53.7%) | 441 (54.0%) | 0.263 |
| **Residual stenosis degree** |  | | | | | 0.089 |
| <25% | 26 (42.6%) | 26 (40.6%) | 21 (38.9%) | 140 (41.8%) | 213 (41.4%) |  |
| 25-49% | 11 (18.0%) | 22 (34.4%) | 18 (33.3%) | 96 (28.7%) | 147 (28.6%) |  |
| 50-75% | 12 (19.7%) | 8 (12.5%) | 13 (24.1%) | 74 (22.1%) | 107 (20.8%) |  |
| >75% | 7 (11.5%) | 6 (9.4%) | 1 (1.9%) | 18 (5.4%) | 32 (6.2%) |  |
| 100% | 5 (8.2%) | 2 (3.1%) | 1 (1.9%) | 7 (2.1%) | 15 (2.9%) |  |
| **Complications** |  | | | | | 0.052 |
| SAH/vessel perforation | 0 (0.0%) | 8 (9.8%) | 5 (5.4%) | 30 (5.5%) | 43 (5.3%) |  |
| Dissection | 3 (3.0%) | 3 (3.7%) | 0 (0.0%) | 13 (2.4%) | 19 (2.3%) |  |
| Femoral/retroperitoneal hematoma | 0 (0.0%) | 2 (2.4%) | 1 (1.1%) | 7 (1.3%) | 10 (1.2%) |  |
| Other | 2 (2.0%) | 7 (8.5%) | 3 (3.3%) | 22 (4.0%) | 34 (4.2%) |  |
| **Post treatment occlusion** |  | | | | | 0.053 |
| Yes | 21 (22.1%) | 11 (13.8%) | 13 (14.6%) | 64 (12.1%) | 109 (13.7%) |  |
| No control | 6 (6.3%) | 10 (12.5%) | 17 (19.1%) | 76 (14.3%) | 109 (13.7%) |  |
| **Reocclusion time** |  | | | | | 0.278 |
| within 24h | 13 (61.9%) | 9 (75.0%) | 9 (69.2%) | 29 (43.9%) | 60 (53.6%) |  |
| 24h-discharge | 7 (33.3%) | 2 (16.7%) | 4 (30.8%) | 31 (47.0%) | 44 (39.3%) |  |
| after discharge | 1 (4.8%) | 1 (8.3%) | 0 (0.0%) | 6 (9.1%) | 8 (7.1%) |  |
| **Clinical worsening due to reocclusion** | 28 (27.7%) | 17 (21.0%) | 6 (6.6%) | 85 (17.1%) | 136 (17.7%) | 0.001 |
| **Hemorrhagic transformation** |  | | | | | 0.162 |
| HI1 | 11 (11.7%) | 1 (1.3%) | 3 (4.1%) | 48 (9.6%) | 63 (8.5%) |  |
| HI2 | 7 (7.4%) | 4 (5.3%) | 4 (5.4%) | 28 (5.6%) | 43 (5.8%) |  |
| PH1 | 1 (1.1%) | 0 (0.0%) | 2 (2.7%) | 18 (3.6%) | 21 (2.8%) |  |
| PH2 | 1 (1.1%) | 1 (1.3%) | 3 (4.1%) | 14 (2.8%) | 19 (2.6%) |  |
| **Symptomatic ICH** | 3 (3.1%) | 5 (6.1%) | 6 (7.8%) | 50 (9.1%) | 64 (7.9%) | 0.206 |
| **Discharge NIHSS, median (IQR)** | 6.0 (2.0, 14.0) | 4.0 (1.8, 14.2) | 8.0 (4.0, 16.0) | 5.0 (2.0, 13.0) | 5.0 (2.0, 14.0) | 0.150 |
| **Discharge mRS, median (IQR)** | 4.0 (2.0, 5.0) | 4.0 (2.0, 5.0) | 4.0 (2.0, 5.0) | 4.0 (2.0, 5.0) | 4.0 (2.0, 5.0) | 0.413 |
| **In-hospital death** | 19 (19.0%) | 14 (16.9%) | 16 (21.6%) | 89 (16.2%) | 138 (17.1%) | 0.658 |
| **90 days mRS, median (IQR)** | 3.5 (2.0, 6.0) | 3.0 (1.0, 5.2) | 4.0 (2.0, 5.0) | 3.0 (1.0, 6.0) | 3.0 (1.0, 6.0) | 0.434 |
| **mRS 0-2** | 42 (42.0%) | 32 (42.1%) | 26 (35.6%) | 229 (43.1%) | 329 (42.2%) | 0.686 |
| **Stroke recurrence within 90 days** | 5 (7.7%) | 0 (0.0%) | 3 (5.7%) | 15 (3.8%) | 23 (4.0%) | 0.165 |
| **Restenosis recurrence within 90 days** |  | | | | | 0.157 |
| No | 23 (54.8%) | 31 (67.4%) | 27 (71.1%) | 197 (65.7%) | 278 (65.3%) |  |
| <25% | 3 (7.1%) | 6 (13.0%) | 6 (15.8%) | 43 (14.3%) | 58 (13.6%) |  |
| 25-50% | 5 (11.9%) | 4 (8.7%) | 2 (5.3%) | 34 (11.3%) | 45 (10.6%) |  |
| >75% | 8 (19.0%) | 3 (6.5%) | 3 (7.9%) | 16 (5.3%) | 30 (7.0%) |  |
| 100% | 3 (7.1%) | 2 (4.3%) | 0 (0.0%) | 10 (3.3%) | 15 (3.5%) |  |
| **Retreatment within 90 days** |  | | | | | 0.100 |
| No | 30 (93.8%) | 34 (97.1%) | 25 (96.2%) | 140 (97.9%) | 229 (97.0%) |  |
| Angioplasty | 2 (6.2%) | 1 (2.9%) | 0 (0.0%) | 3 (2.1%) | 6 (2.5%) |  |
| Re-stenting | 0 (0.0%) | 0 (0.0%) | 1 (3.8%) | 0 (0.0%) | 1 (0.4%) |  |

**Supplementary Table 3.** Outcomes according to intraprocedural stent occlusion.

|  | **Intraprocedural stent occlusion** | | |
| --- | --- | --- | --- |
|  | **Yes** | **No** | **p-value** |
| **Final TICI** |  | | |
| TICI 0 | 6 (12.5%) | 31 (3.8%) | 0.060 |
| TICI 1 | 0 (0.0%) | 5 (0.6%) |  |
| TICI 2a | 4 (8.3%) | 45 (5.5%) |  |
| TICI 2b | 7 (14.6%) | 173 (21.1%) |  |
| TICI 2c | 4 (8.3%) | 102 (12.4%) |  |
| TICI 3 | 27 (56.2%) | 465 (56.6%) |  |
| **Successful recanalization** |  | | |
| TICI 2b-3 | 38 (79.2%) | 740 (90.1%) | 0.016 |
| TICI 2c-3 | 31 (64.6%) | 567 (69.1%) | 0.515 |
| **Treatment of intraprocedural occlusion** | 22 (28.6%) | 55 (71.4%) | 0.001 |
| **Residual ICAS** | 19 (39.6%) | 441 (54.0%) | 0.052 |
| **Residual stenosis degree** |  | | |
| <25% | 11 (42.3%) | 213 (41.4%) | 0.016 |
| 25-49% | 5 (19.2%) | 147 (28.6%) |  |
| 50-75% | 4 (15.4%) | 107 (20.8%) |  |
| >75% | 2 (7.7%) | 32 (6.2%) |  |
| 100% | 4 (15.4%) | 15 (2.9%) |  |
| **Complications** |  | | |
| SAH/vessel perforation | 5 (10.2%) | 43 (5.3%) | 0.117 |
| Dissection | 3 (6.1%) | 19 (2.3%) |  |
| Femoral/retroperitoneal hematoma | 0 (0.0%) | 10 (1.2%) |  |
| Other | 0 (0.0%) | 34 (4.2%) |  |
| **Post treatment occlusion** |  | | |
| Yes | 8 (16.3%) | 109 (13.7%) | 0.858 |
| No control | 6 (12.2%) | 109 (13.7%) |  |
| **Reocclusion time** |  | | |
| within 24h | 8 (100.0%) | 60 (53.6%) | 0.038 |
| 24h-discharge | 0 (0%) | 44 (39.3%) |  |
| after discharge | 0 (0%) | 8 (7.1%) |  |
| **Clinical worsening due to reocclusion** | 9 (19.6%) | 136 (17.7%) | 0.743 |
| **Hemorrhagic transformation** |  | | |
| HI1 | 3 (6.4%) | 63 (8.5%) | 0.023 |
| HI2 | 2 (4.3%) | 43 (5.8%) |  |
| PH1 | 0 (0.0%) | 21 (2.8%) |  |
| PH2 | 5 (10.6%) | 19 (2.6%) |  |
| **Symptomatic ICH** | 6 (12.2%) | 64 (7.9%) | 0.285 |
| **Discharge NIHSS, median (IQR)** | 6.0 (2.5, 15.5) | 5.0 (2.0, 14.0) | 0.285 |
| **Discharge mRS, median (IQR)** | 4.0 (3.0, 6.0) | 4.0 (2.0, 5.0) | 0.032 |
| **In-hospital death** | 13 (26.5%) | 138 (17.1%) | 0.094 |
| **90 days mRS, median (IQR)** | 4.0 (2.0, 6.0) | 3.0 (1.0, 6.0) | 0.124 |
| **mRS 0-2** | 16 (33.3%) | 329 (42.2%) | 0.228 |
| **Stroke recurrence within 90 days** | 1 (2.9%) | 23 (4.0%) | 0.732 |

**Supplementary Table 4.** Univariable and multivariable logistic regression analysis for intraprocedural stent occlusion.

|  | **Univariable** | | | **Multivariable** | | |
| --- | --- | --- | --- | --- | --- | --- |
| **Variables** | **OR** | **95% CI** | **p-value** | **OR** | **95% CI** | **p-value** |
| Oral DAPT vs sAPT | 0.44 | 0.19 – 1.05 | 0.073 | 0.48 | 0.15 – 1.57 | 0.380 |
| Cangrelor vs sAPT | 0.15 | 0.05 – 0.45 | <0.001 * | 0.17 | 0.04 – 0.79 | 0.017* |
| GPi vs sAPT | 0.09 | 0.04 – 0.19 | <0.001 * | 0.12 | 0.05 – 0.30 | <0.001* |
| Cangrelor vs oral dAPT | 0.34 | 0.11 – 1.10 | 0.086 | 0.35 | 0.06 – 1.89 | 0.375 |
| GPi vs oral dAPT | 0.21 | 0.09 – 0.48 | <0.001* | 0.25 | 0.08 – 0.78 | 0.009* |
| IV APT vs oral dAPT | 0.27 | 0.11 – 0.64 | <0.001 * | 0.30 | 0.09-1.01 | 0.053 |
| GPi vs Cangrelor | 0.61 | 0.21 – 1.75 | 0.615 | 0.72 | 0.16 – 3.30 | 0.944 |
| Number of passes pre-stenting | - | - | - | 0.970 | 0.806 – 1.167 | 0.747 |
| Age | - | - | - | 0.983 | 0.960 – 1.007 | 0.168 |
| Sex (Male) | - | - | - | 0.473 | 0.255 – 0.877 | 0.018 * |
| Pre-stroke mRS | - | - | - | 1.137 | 0.840 – 1.538 | 0.406 |
| Baseline NIHSS | - | - | - | 1.005 | 0.969 – 1.043 | 0.781 |
| IVT | - | - | - | 1.270 | 0.646 – 2.499 | 0.489 |
| ASPECTS | - | - | - | 0.995 | 0.802 – 1.233 | 0.961 |
| Onset-to-recanalization | - | - | - | 1.000 | 0.999 – 1.000 | 0.675 |
| Proximal occlusion | - | - | - | 0.783 | 0.320 – 1.918 | 0.593 |
| Posterior circulation | - | - | - | 0.809 | 0.387 – 1.690 | 0.573 |
| Tandem occlusion | - | - | - | - | 0.000 – Inf | 0.984 |
| Recanalization pre-stenting | - | - | - | 1.212 | 0.652 – 2.252 | 0.544 |

**Supplementary Table 5.** Univariable and multivariable logistic regression analysis for TICI 2c-3.

|  | **Univariable** | | | **Multivariable** | | |
| --- | --- | --- | --- | --- | --- | --- |
| **Variables** | **OR** | **95% CI** | **p-value** | **OR** | **95% CI** | **p-value** |
| Oral DAPT vs sAPT | 1.20 | 0.55 – 2.61 | 0.934 | 0.78 | 0.26 – 2.37 | 0.941 |
| Cangrelor vs sAPT | 2.97 | 1.29 – 6.82 | 0.005* | 5.14 | 1.45 – 18.28 | 0.005* |
| GPi vs sAPT | 1.88 | 1.07 – 3.30 | 0.022* | 2.26 | 1.03 – 4.97 | 0.041* |
| Cangrelor vs oral dAPT | 2.48 | 1.03 – 5.99 | 0.040* | 6.56 | 1.71 – 25.13 | 0.002* |
| GPi vs oral dAPT | 1.57 | 0.83 – 2.96 | 0.257 | 2.88 | 1.14 – 7.26 | 0.018* |
| IV APT vs oral dAPT | 1.97 | 0.99-3.91 | 0.053 | 4.35 | 1.57-12.09 | 0.001* |
| GPi vs Cangrelor | 0.63 | 0.31 – 1.27 | 0.328 | 0.44 | 0.15 – 1.30 | 0.208 |
| Number of passes pre-stenting | - | - | - | 0.696 | 0.602 – 0.805 | <0.001* |
| Age | - | - | - | 1.009 | 0.991 – 1.027 | 0.316 |
| Sex (Male) | - | - | - | 1.018 | 0.646 – 1.606 | 0.939 |
| Pre-stroke mRS | - | - | - | 0.675 | 0.542 – 0.840 | <0.001* |
| Baseline NIHSS | - | - | - | 0.989 | 0.963 – 1.015 | 0.401 |
| IVT | - | - | - | 1.821 | 1.085 – 3.054 | 0.024* |
| ASPECTS | - | - | - | 1.119 | 0.964 – 1.299 | 0.138 |
| Onset-to-recanalization | - | - | - | 1.001 | 1.000 – 1.001 | 0.005* |
| Proximal occlusion | - | - | - | 1.005 | 0.527 – 1.918 | 0.988 |
| Posterior circulation | - | - | - | 1.184 | 0.701 – 2.002 | 0.528 |
| Tandem occlusion | - | - | - | 1.401 | 0.518 – 3.790 | 0.507 |
| Recanalization pre-stenting | - | - | - | 2.782 | 1.748 – 4.426 | <0.001* |

**Supplementary Table 6.** Univariable and multivariable logistic regression analysis for occlusion within 24 hours.

|  | **Univariable** | | | **Multivariable** | | |
| --- | --- | --- | --- | --- | --- | --- |
| **Variables** | **OR** | **95% CI** | **p-value** | **OR** | **95% CI** | **p-value** |
| Oral DAPT vs SAPT-ASA | 1.23 | 0.32 – 4.78 | 0.980 | 4.94 | 0.71 – 34.18 | 0.146 |
| Cangrelor vs SAPT-ASA | 1.13 | 0.29 – 4.38 | 0.996 | 2.13 | 0.26 – 17.58 | 0.790 |
| GPi vs SAPT-ASA | 0.42 | 0.14 – 1.28 | 0.185 | 1.23 | 0.23 – 6.54 | 0.989 |
| Cangrelor vs oral dAPT | 0.92 | 0.24 – 3.60 | 0.999 | 0.43 | 0.07 – 2.71 | 0.639 |
| GPi vs oral dAPT | 0.34 | 0.11 – 1.05 | 0.067* | 0.25 | 0.06 – 0.99 | 0.047* |
| IV APT vs oral dAPT | 0.56 | 0.18 – 1.72 | 0.540 | 0.33 | 0.08 – 1.36 | 0.181 |
| GPi vs Cangrelor | 0.37 | 0.12 – 1.14 | 0.104 | 0.58 | 0.12 – 2.84 | 0.808 |
| Number of passes pre-stenting | - | - | - | 0.97 | 0.76 – 1.23 | 0.778 |
| Age | - | - | - | 1.00 | 0.97 – 1.03 | 0.892 |
| Sex (Male) | - | - | - | 0.86 | 0.38 – 1.91 | 0.704 |
| Pre-stroke mRS | - | - | - | 0.78 | 0.47 – 1.28 | 0.329 |
| Baseline NIHSS | - | - | - | 1.03 | 0.98 – 1.08 | 0.212 |
| IVT | - | - | - | 1.08 | 0.45 – 2.61 | 0.865 |
| ASPECTS | - | - | - | 0.97 | 0.75 – 1.26 | 0.819 |
| Onset-to-recanalization | - | - | - | 1.00 | 0.99 – 1.00 | 0.331 |
| Proximal occlusion | - | - | - | 1.31 | 0.35 – 4.86 | 0.691 |
| Posterior circulation | - | - | - | 0.62 | 0.23 – 1.63 | 0.330 |
| Tandem occlusion | - | - | - | - | 0.000 – Inf | 0.985 |
| Recanalization pre-stenting | - | - | - | 1.46 | 0.65 – 3.27 | 0.357 |

**Supplementary Table 7.** Univariable and multivariable logistic regression analysis for 3 months mRS.

|  | **Univariable** | | | **Multivariable** | | |
| --- | --- | --- | --- | --- | --- | --- |
| **Variables** | **OR** | **95% CI** | **p-value** | **OR** | **95% CI** | **p-value** |
| Oral dAPT vs sAPT | 1.00 | 0.46 – 2.20 | 1.000 | 0.66 | 0.20 – 2.19 | 0.806 |
| Cangrelor vs sAPT | 0.76 | 0.34 – 1.71 | 0.824 | 0.71 | 0.21 – 2.48 | 0.896 |
| GPi vs sAPT | 1.05 | 0.60 – 1.84 | 0.997 | 0.73 | 0.31 – 1.71 | 0.775 |
| Cangrelor vs oral dAPT | 0.76 | 0.32 – 1.79 | 0.842 | 1.08 | 0.29 – 4.08 | 0.999 |
| GPi vs oral dAPT | 1.04 | 0.55 – 1.96 | 0.998 | 1.11 | 0.42 – 2.90 | 0.993 |
| IV APT vs oral dAPT | 0.89 | 0.45-1.76 | 0.971 | 1.09 | 0.38-3.10 | 0.996 |
| GPi vs Cangrelor | 1.37 | 0.71 – 2.65 | 0.605 | 1.02 | 0.36 – 2.87 | 1.000 |
| Number of passes pre-stenting | - | - | - | 0.825 | 0.712 – 0.957 | 0.011 |
| Age | - | - | - | 0.962 | 0.945 – 0.980 | <0.001 |
| Sex (Male) | - | - | - | 0.792 | 0.498 – 1.259 | 0.324 |
| Pre-stroke mRS | - | - | - | 0.596 | 0.459 – 0.774 | <0.001 |
| Baseline NIHSS | - | - | - | 0.918 | 0.891 – 0.946 | <0.001 |
| IVT | - | - | - | 1.067 | 0.636 – 1.790 | 0.808 |
| ASPECTS | - | - | - | 1.155 | 0.984 – 1.356 | 0.079 |
| Onset-to-recanalization | - | - | - | 1.000 | 0.999 – 1.000 | 0.286 |
| Proximal occlusion | - | - | - | 1.415 | 0.720 – 2.778 | 0.314 |
| Posterior circulation | - | - | - | 0.664 | 0.404 – 1.092 | 0.107 |
| Tandem occlusion | - | - | - | 0.454 | 0.163 – 1.259 | 0.130 |
| Recanalization pre-stenting | - | - | - | 2.351 | 1.499 – 3.688 | <0.001 |

**Supplementary Table 8.** Univariable and multivariable logistic regression analysis for all HT and sICH.

|  | **Univariable** | | | **Multivariable** | | |
| --- | --- | --- | --- | --- | --- | --- |
| **Variables** | **OR** | **95% CI** | **p-value** | **OR** | **95% CI** | **p-value** |
| **All Hemorrhagic Transformations** | | | | | | |
| Oral dAPT vs sAPT | 0.31 | 0.09 – 1.11 | 0.085 | 0.22 | 0.04 - 1.08 | 0.068 |
| Cangrelor vs sAPT | 0.72 | 0.25 – 2.01 | 0.844 | 0.71 | 0.20 – 2.48 | 0.895 |
| GPi vs sAPT | 0.99 | 0.49 – 2 | 1 | 0.66 | 0.29 – 1.48 | 0.548 |
| Cangrelor vs oral dAPT | 2.28 | 0.59 - 8.77 | 0.383 | 3.14 | 0.56 – 17.61 | 0.312 |
| GPi vs oral dAPT | 3.15 | 1.03 - 9.61 | 0.041* | 2.91 | 0.69 – 12.25 | 0.217 |
| IV APT vs oral dAPT | 2.68 | 0.84 - 8.56 | 0.125 | 3.02 | 0.68 – 13.41 | 0.218 |
| GPi vs Cangrelor | 1.37 | 0.59 - 3.21 | 0.756 | 0.92 | 0.30 – 2.76 | 0.997 |
| Number of passes pre-stenting | - | - | - | 1.12 | 0.97 – 1.30 | 0.111 |
| Age | - | - | - | 0.99 | 0.97 – 1.01 | 0.550 |
| Sex (Male) | - | - | - | 0.67 | 0.40 – 1.10 | 0.119 |
| Pre-stroke mRS | - | - | - | 1.00 | 0.78 – 1.28 | 0.968 |
| Baseline NIHSS | - | - | - | 1.01 | 0.98 – 1.04 | 0.305 |
| IVT | - | - | - | 0.92 | 0.52 – 1.60 | 0.770 |
| ASPECTS | - | - | - | 0.95 | 0.80 – 1.12 | 0.547 |
| Onset-to-recanalization | - | - | - | 1.00 | 1.00 – 1.00 | 0.765 |
| Proximal occlusion | - | - | - | 1.35 | 0.61 – 2.98 | 0.454 |
| Posterior circulation | - | - | - | 0.89 | 0.50 – 1.59 | 0.716 |
| Tandem occlusion | - | - | - | 1.38 | 0.47 – 4.07 | 0.550 |
| Recanalization (TICI 2b–3) | - | - | - | 1.25 | 0.76 – 2.06 | 0.364 |
| **Symptomatic ICH** | | | | | | |
| Oral dAPT vs sAPT | 2.06 | 0.31 – 13.69 | 0.756 | 2.09 | 0.18 – 23.76 | 0.859 |
| Cangrelor vs sAPT | 2.68 | 0.42 – 16.85 | 0.507 | 4.99 | 0.52 – 47.73 | 0.254 |
| GPi vs sAPT | 3.17 | 0.68 – 14.72 | 0.213 | 2.92 | 0.43 – 19.97 | 0.471 |
| Cangrelor vs oral dAPT | 1.30 | 0.26 – 6.41 | 0.973 | 2.38 | 0.31 – 18.56 | 0.689 |
| GPi vs oral dAPT | 1.54 | 0.45 – 5.28 | 0.798 | 1.40 | 0.26 – 7.41 | 0.954 |
| IV APT vs oral dAPT | 1.42 | 0.38-5.23 | 0.900 | 1.82 | 0.32-10.33 | 0.803 |
| GPi vs Cangrelor | 1.18 | 0.38 – 3.71 | 0.981 | 0.59 | 0.14-2.44 | 0.763 |
| Number of passes pre-stenting | - | - | - | 1.132 | 0.916 – 1.398 | 0.252 |
| Age | - | - | - | 0.998 | 0.970 – 1.027 | 0.876 |
| Sex (Male) | - | - | - | 0.972 | 0.458 – 2.063 | 0.941 |
| Pre-stroke mRS | - | - | - | 0.867 | 0.577 – 1.302 | 0.491 |
| Baseline NIHSS | - | - | - | 1.035 | 0.993 – 1.079 | 0.104 |
| IVT | - | - | - | 0.969 | 0.430 – 2.187 | 0.940 |
| ASPECTS | - | - | - | 1.119 | 0.865 – 1.449 | 0.392 |
| Onset-to-recanalization | - | - | - | 1.000 | 0.999 – 1.001 | 0.475 |
| Proximal occlusion | - | - | - | 1.232 | 0.400 – 3.796 | 0.716 |
| Posterior circulation | - | - | - | 0.661 | 0.277 – 1.577 | 0.352 |
| Tandem occlusion | - | - | - | 1.714 | 0.461 – 6.379 | 0.422 |
| Recanalization (TICI 2b–3) | - | - | - | 1.269 | 0.604 – 2.668 | 0.530 |

**Supplementary Table 9.** Standard mean difference after IPTW to compare GPi and Cangrelor.

| **Variable** | **Mean SMD** |
| --- | --- |
| Age | -0.0671 |
| Sex, male | 0.0038 |
| Pre-stroke mRS | -0.0245 |
| Baseline NIHSS | 0.0111 |
| IVT | -0.0136 |
| ASPECTS | -0.0617 |
| Onset to Recanalization | 0.0003 |
| Proximal vessel occlusion | 0.0201 |
| Posterior vessel occlusion | -0.0147 |
| Tandem Occlusion | -0.0022 |
| Recanalization (TICI 2b-3) | -0.0005 |
| Number of passes before senting | 0.0182 |
